# Evaluation of the Breed Composition of Pork via Population Structure Analysis in Pigs

**DOI:** 10.3390/ani14233489

**Published:** 2024-12-03

**Authors:** Qing Lin, Shuqi Diao, Xinyou Chen, Jinshi Du, Jiaxuan Wu, Xinshuo Zhang, Xiaohong Liu, Jiaqi Li, Zhe Zhang

**Affiliations:** 1 State Key Laboratory of Swine and Poultry Breeding Industry, Guangdong Provincial Key Laboratory of Agro-Animal Genomics and Molecular Breeding, College of Animal Science, South China Agricultural University, Guangzhou 510642, China; qing_lin1996@126.com (Q.L.);; 2 State Key Laboratory of Biocontrol, School of Life Sciences, Sun Yat-Sen University, Guangzhou 510275, China

**Keywords:** ancestry identification, breed composition, meat quality, indigenous breed, crossbreed, structure analysis

## Abstract

The quality of pork meat directly influences the price and consumption. The genetic improvement of pigs has mainly focused on high productive efficiency, which has resulted in poor meat quality. Crossbreeds containing commercial and indigenous breeds could improve the meat quality, but identifying breed composition was difficult because of the lack of an ancestry reference panel. Therefore, we first constructed an abundant reference panel and convenient pipeline to identify ancestry/breed composition. The ancestry reference panel consisted of 517 reliable individuals, including three commercial breeds (Duroc, Landrace, and Yorkshire) and 38 indigenous Chinese breeds. The nature of the reference panel showed that the European domestic breed (EUD) and Asian domestic breed (ASD) were distinctly divided into two clusters. The evaluation of ancestry identification revealed that the reference panel performed well in identifying EUD and ASD ancestry proportions for commercial breeds, indigenous breeds, and crossbreeds. In addition, the ancestry reference panel also performed excellently in identifying breed composition for 3 commercial and 38 indigenous breeds. Specifically, the reference panel showed the outstanding identification of breed composition for crossbred individuals. These results suggested that the ancestry reference panel and convenient pipeline played a good role in identifying breed composition for pigs.

## Simple Summary

The quality of pork meat is partially impacted by the breed composition, while the breed composition is difficult to deconvolute because of the lack of a reference panel and comprehensive pipeline. In this study, we established the ancestry reference panel and developed a comprehensive pipeline to identify the breed composition for purebred and crossbred pigs. In summary, the ancestry reference panel contains 3 commercial and 38 indigenous breeds for breed identification. We found that the workflow could favorably identify the breed information for pure breeds. In addition, it could also favorably deconvolute the breed composition for cross breeds in pigs. We suggest that the workflow could be utilized to identify the breed composition and evaluate the quality of pork meat from pigs.

## 1. Introduction

The quality of pork meat directly influences the price and consumption, while the breed composition of the pig is partially reflected in the meat quality and preference of consumers. The genetic improvement of pigs has focused on high productive efficiency, including high lean meat percentage and low thickness of backfat [1], which has gradually resulted in poor meat quality because of the decrease of intramuscular fat content and ignorance of the chemical components [2,3]. With the development of social economies, most consumers demand higher eating quality of pork meat, such as the intramuscular fat content and taste [4]. The indigenous breeds in China have excellent meat quality, while their growth efficiency is lower than that of commercial breeds [5,6]. Previous studies have demonstrated that the crossbreed between commercial and indigenous Chinese breeds could favorably improve the quality of pork meat [7,8]. The pork circulating in the market usually originates from crossbreeds because of heterosis. Therefore, the identification of breed composition for the pig could partially reflect the quality of the meat; though it is hard to identify the breed information for meat products, especially the breed composition for crossbreeds.

Genetic features and diversity could be explored by the genome-wide scan of different breeds, which favorably represents the characteristics of corresponding breeds. Diao et al. [9] found the evident differentiation of genetic architecture and special signature of selection between indigenous breeds from South China and Duroc pigs. In addition, a population genetic analysis demonstrated significant genetic differentiation and a moderate mixture among the indigenous Chinese meta-populations [10]. Wang et al. [11] also identified the breed-specific genetic signatures through a genome-wide scan and preferably predicted the breed information using a machine-learning classification model in eight chicken breeds. In addition, the copy number variation (CNV) also played an essential role in forming breed characteristics between domestic and wild pigs [12]. Generally, genetic variations, such as single nuclear polymorphism (SNP) and CNV, effectively reflect the formation of breed characteristics, which might be leveraged to identify the breed information.

Breed identification could accurately identify the commercial and indigenous breeds; while evaluating the breed composition for crossbreeds is difficult. For example, the tag single nucleotide polymorphisms (SNPs) with machine learning methods demonstrated excellent performance in breed identification [13,14]. The selection of ancestry informative markers (AIMs) also performed good identification of breed composition, though this strategy is usually used to identify the ancestry proportion of regional populations/breeds. For example, Bertolini et al. [15] found 96 AIMs performed well in identification for six dairy cattle breeds. Liang et al. [16] selected 129 AIMs to estimate the ancestry proportion, and the results demonstrated a high correlation with the estimates of low-density variants in pigs. Recent analysis indicated that a large reference panel could feasibly identify the pig breeds, including purebred and crossbreeds [17]. Furthermore, a previous study showed that three reference panels could effectively capture individual variations in admixture proportions in humans [18]. These findings highlight the importance of constructing an ancestry reference panel to accurately identify the ancestry proportions of both purebred and crossbred pigs.

Therefore, we first constructed an ancestry reference panel and convenient pipeline for ancestry identification using the Pig Genomics Reference Panel (PGRP v1) [19]. We then conducted a population structure analysis to select reliable individuals, including a principal component analysis (PCA) for commercial breeds and a population structure analysis for indigenous Chinese breeds. To further evaluate the identification performance of the reference panel, we implemented ancestry identification, including (1) European domestic breed (EUD) and Asian domestic breed (ASD) proportion identification and (2) breed proportion identification.

## 2. Materials and Methods

### 2.1. Genotypes

We extracted the genotype data from the Pig Genome Reference Panel (PGRP v1) [19] according to the “Zhongxin-I” Porcine Breeding Chip 50 K (Beijing Compass Agritechnology Co., Ltd., Beijing, China), including 1602 individuals with 34,386 variants. Next, we obtained the LD-prune SNPs and we conducted the linkage disequilibrium pruning using PLINK v1.90 [20] with parameter ‘--indep-pairwise 50 10 0.5’ that indicated the variants with high LD (r^2^ > 0.5) in 50 variants windows were pruned until no such pairs remain. Finally, 1602 individuals with 24,595 common variants were utilized for the downstream analysis.

### 2.2. The Selection of Reliable Individuals for Ancestry Reference Panel

#### 2.2.1. The Selection of Reliable Individuals in Commercial Breeds

To ensure the accuracy of ancestry identification of the reference panel, we carried out a principal component analysis (PCA) for each commercial breed, including Duroc (D), Landrace (L), Yorkshire (Y), Berkshire (B), Hampshire (H) and Piétrain (P), to select the reliable individuals using PLINK v1.90 and visualized via ggplot2 R package [20].

We selected the 30 credible individuals according to the parameters for Duroc (PC1 < 0 and PC2 < −0.05), Landrace (PC1 < 0 and PC2 < 0) and Yorkshire (PC1 > 0 and PC2 < 0) for each commercial breed (Figure S1).

#### 2.2.2. The Selection of Reliable Individuals in Indigenous Breeds

We conducted a population structure analysis to choose the reliable individuals for each indigenous Chinese breed. We first combined the genotype of selected D/L/Y individuals (90 individuals in total) with each indigenous breed (sample size for each indigenous breed in Table S1) and processed the structure analysis using ADMIXTURE v1.3.0 [21] with K = 4. Based on the result of structure analysis, we then calculated the average ancestry proportion of all individuals from indigenous breeds for each K (K_1_, K_2_, K_3_, and K_4_). After that, we defined the K_i_ with the maximum average ancestry proportion as the indigenous breed-specific proportion (P_IB_). Next, we defined the indigenous breed with average P_IB_ < 0.50 as the mixed indigenous breeds because of high commercial ancestry proportion. We removed these mixed indigenous breeds and finally 38 indigenous breeds were maintained for further analysis. Based on the selected indigenous breeds, we further selected the individuals with P_IB_ > 0.80 as the reliable individuals in each indigenous breed. Finally, 427 reliable individuals from 38 indigenous breeds were kept to construct the reference panel. The details of sample size information about the reference panel are in Table S1.

### 2.3. The Genetic Architecture of Ancestry Reference Panel

To explore the population architecture of the reference panel, we implemented three types of population structure analysis for the reference panel. We first calculated the genetic distance between individuals using PLINK v1.90 with parameter “--genome”. After that, we transferred the results of genetic distance into .meg format using R v4.2.1 [21] and constructed a neighbor-joining tree (.nwk format) using MEGA v11.0.13 [22]. Then, we visualized the neighbor-joining tree by ggtree R package [23]. We next produced a PCA using PLINK v1.90 with parameter “--pca 517” to observe the distribution of ASD/EUD group in the reference panel. Finally, we carried out the structure analysis with K = 2 using ADMIXTURE v1.3.0 to identify the composition of ancestry proportion in the reference panel.

### 2.4. The Identification of Ancestry Proportion for Validation Set

#### 2.4.1. The Ancestry Identification of EUD and ASD Proportion for Validation Set

To evaluate the performance of the reference panel in ancestry identification of EUD and ASD proportion, we first constructed a validation set, including 514 individuals with clear labels. For the purebred individuals, we focused on the individuals with clear breed information. For the crossbred individuals, we took the individuals with explicit cross-breed information (such as D × (L × Y) and D × DNXE) into account in the ancestry reference panel. We combined the genotype of the reference panel with each valid individual to conduct the structure analysis with K = 2 using ADMIXTURE v1.3.0. We then divided the reference panel into two groups (ASD/EUD group, Table S1). Next, we calculated the average ancestry proportion and defined the maximum proportion as the corresponding EUD/ASD-specific proportion for each group. We assigned the information on the EUD/ASD-specific proportion to each valid individual. Finally, we evaluated the performance of EUD/ASD ancestry identification on the basis of the EUD/ASD-specific proportion. We utilized the estimated ancestry proportion of the candidates with the corresponding breed information to evaluate the performance of the ancestry reference panel.

#### 2.4.2. The Ancestry Identification of Commercial and Indigenous Breeds for Validation Set

To assess the performance of the reference panel in ancestry identification of commercial and indigenous breeds, we combined the genotype of selected D/L/Y individuals (90 individuals) and each indigenous breed with each valid individual to produce the structure analysis with K = 5 using ADMIXTURE v1.3.0. We grouped the individuals from the reference panel into four groups (including D/L/Y/Indigenous group) according to the breed information and calculated the mean of each ancestry proportion for each group. We also defined the D/L/Y/Indigenous-specific proportion according to the maximum of the corresponding proportion. In addition, the remaining component was defined as the Unknown-specific proportion. Finally, we obtained the 38 ancestry proportion results with breed-specific labels for each valid individual. To further select the credible validation of individuals, we first computed each proportion’s average and standard error (SE) from 38 ancestry proportion results. We removed the valid individuals that had more than 2 breed-specific proportion of SE > 0.2. The research data and analyzed pipeline were reposited at https://github.com/SCAU-AnimalGenetics/Ancestry-identification (accessed on 20 April 2023).

## 3. Results

### 3.1. Overview of Workflow

**Figure 1:**
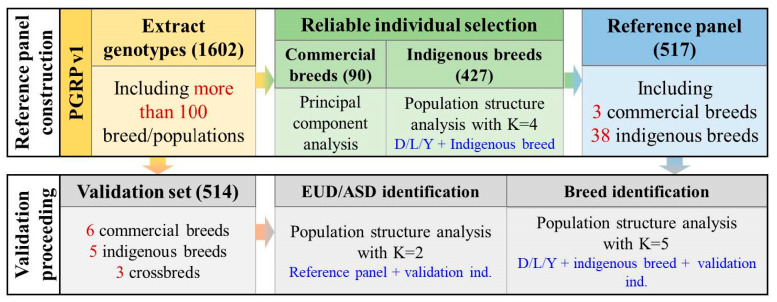
Overview of the ancestry identification workflow. The numbers located in the brackets represents the sample size. PGRP: Pig Genomics Reference Panel; EUD: European domestic breed; ASD: Asian domestic breed; D: Duroc; L: Landrace; Y: Yorkshire.

We constructed an ancestry reference panel and convenient pipeline for ancestry identification (Figure 1) using the Pig Genomics Reference Panel (PGRP v1) to identify the ancestry proportion of the individuals with unknown breed information. Initially, we extracted the low-density genotype from the whole genome sequence data. Then, a population structure analysis was performed to select reliable individuals. For commercial breeds (including Duroc, Landrace, and Yorkshire), we produced a PCA and chose 30 reliable individuals for each breed (Methods). For indigenous breeds, we combined D/L/Y and each indigenous breed to process a population structure analysis on selected reliable individuals. Finally, we established a validation set (including 514 individuals) to evaluate the identification performance of the reference panel, including (1) EUD/ASD proportion identification and (2) breed proportion identification.

### 3.2. The Selection of Reliable Individuals and Nature of Ancestry Reference Panel

**Figure 2:**
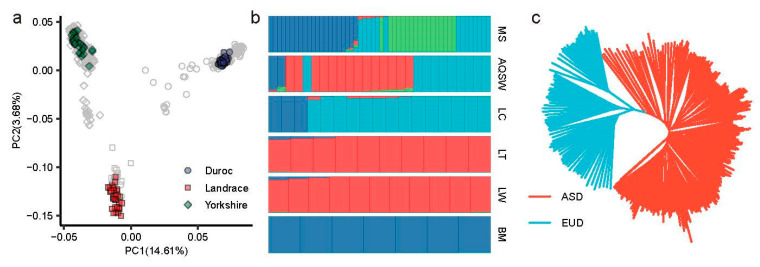
The selection of reliable individuals and nature of reference panel. (**a**) The principal component analysis for Duroc, Landrace, and Yorkshire pigs. The circle, rectangle and diamond points represent Duroc, Landrace and Yorkshire, respectively. Colored points indicate the selected individuals. (**b**) The structure analysis with K = 4 for the Duroc/Landrace/Yorkshire with each indigenous breed. Each bar represents the structure results of indigenous breed plotting by ggplot2 R package. (**c**) The neighbor-joining tree for reference panel plotting by ggtree R package. Blue and red lines indicate the EUD and ASD groups, respectively. The matching of abbreviation and full name of breeds see Table S1 in detail. MS, Meishan; AQSW: An-qing six white; LC, Luchuan; LT, Lantang; LW, Laiwu; BM, Bamei; ASD, Asian domestic breed; EUD, European domestic breed.

We implemented a population structure analysis to construct an ancestry reference panel for ancestry identification. For the commercial breeds, the PCA showed that three commercial breeds distinctly separated into three clusters (Figure 2a). However, several individuals were misassigned to different breed clusters, potentially due to mislabeling. To refine our selection of credible individuals for commercial breeds, we again performed PCA and identified 30 individuals representing each breed’s first and second principal components (Figure S1). The selected individuals were found to cluster appropriately within their respective breed groups (Figure 2a).

For the indigenous breeds, we carried out a population structure analysis and defined the indigenous breed-specific proportion (P_IB_) for each indigenous breed (Table S2, Method). We filtered out the indigenous breed with an average of P_IB_ < 0.50, the cultivated breeds and mixed indigenous breeds, such as Meishan (MS) and Anqing six-end-white (AQSW) (Figure 2b). Then, we retained the individuals with P_IB_ > 0.80 as the reliable individuals for each indigenous breed (Figure 2b), such as Luchuan (LC), Laiwu (LW), and Bamei (BM). Finally, 427 reliable individuals from 38 indigenous breeds were involved in the reference panel (Table S1).

We further implemented three population structure analyses to understand the nature of the reference panel. Firstly, the PCA showed that the EUD group was distinctly separated from the ASD group (Figure S2a). We also conducted a neighbor-joining tree analysis, which revealed that the reference panel was divided into two clusters (Figure 2c). The population structure analysis indicated two proportions for the EUD and ASD group (Figure S2b). These results demonstrated the strong capacity of the reference panel in identifying ancestry proportions.

### 3.3. The Evaluation of ASD/EUD Composition for Validation Set

**Figure 3:**
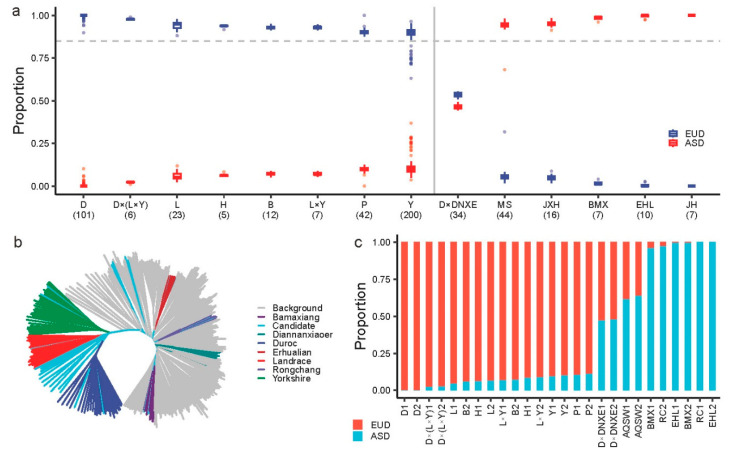
The performance of ancestry identification of ASD or EUD groups. (**a**) The boxplot indicated the ancestry proportion of EUD and ASD groups. The *x*-axis indicates the commercial and indigenous breeds. (**b**) The neighbor-joining tree is for reference panel and 26 valid individuals. The colors indicate different breeds. (**c**) An example of the identification of EUD/ASD proportion for 26 valid individuals. The *x*-axis indicates the purebred or crossbreed individuals. D, Duroc; L, Landrace; Y, Yorkshire; H, Hampshire; B, Berkshire; P, Piétrain; D × (L × Y), Duroc × (Landrace × Yorkshire); L × Y, Landrace × Yorkshire; D × DNXE, Duroc × Diannanxiaoer; JXH, Jiaxinghei; BMX, Bamaxiang; EHL, Erhualian; JH, Jinhua.

To evaluate the performance of ancestry identification for ASD and EUD groups, we utilized the validation set (including 514 individuals with clear labels) to process the population structure analysis (Method). The results demonstrated the proper performance of ancestry identification (Figure 3a), which showed the outstanding performance of ancestry identification for commercial breeds, indigenous breeds, and crossbreeds.

Further, we randomly selected 26 valid individuals to explore the performance of ancestry identification. The neighbor-joining tree showed that valid individuals were located in the corresponding breed/population clusters, such as Erhualian (EHL) and Bamaxiang (BMX) (Figure 3b).

For the common pure breeds, the performance of ancestry identification showed good identification for D, L, and Y, even breeds not included in the reference panel, including Piétrain (P), Hampshire (H), and Berkshire (B) (Figure 3c). It also performed good identification for indigenous breeds, such as Erhualian, Bamaxiang, and Rongchang (RC) (Figure 3c). In addition, we found that the Anqing six-end-white (AQSW) contained about one-third EUD proportion (Figure 3c), which indicated that that AQSW might recently have been mixed with commercial breeds in artificial selection.

On the other hand, the reference panel also demonstrated outstanding identification for crossbreeds. For instance, the D × (L × Y) and L × Y contained a high EUD ancestry proportion, while the Duroc × Diannanxiaoer (D × DNXE) consisted of half of EUD and ASD ancestry proportion (Figure 3c). These results indicated that the reference panel could well identify the EUD and ASD ancestry proportions.

### 3.4. The Evaluation of Breed Composition for Validation Set

**Figure 4:**
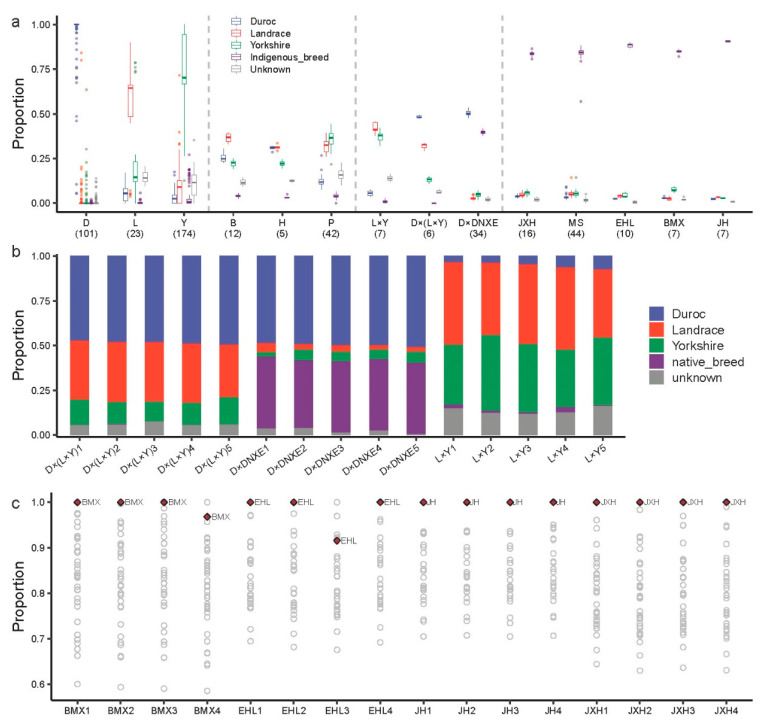
The performance of ancestry identification for breeds. (**a**) The boxplot indicated the ancestry proportion of breed identification. The *x*-axis indicates the commercial and indigenous breeds. Boxplots depict the median value as the center, first, and third quartiles as box boundaries, and whiskers extending 1.5-times the inter-quartile range, individually showing points beyond this region. (**b**) The example of breed identification for 15 crossbred individuals. The colors represent the breed information the same as the legend of panel (**a**). (**c**) The example of breed identification for 16 indigenous breed individuals. Each point represents indigenous breed information. The red point is the corresponding indigenous breed with the valid individuals. D, Duroc; L, Landrace; Y, Yorkshire; H, Hampshire; B, Berkshire; P, Piétrain; D × (L × Y), Duroc × (Landrace × Yorkshire); L × Y, Landrace × Yorkshire; D × DNXE, Duroc × Diannanxiaoer; JXH, Jiaxinghei; BMX, Bamaxiang; EHL, Erhualian; JH, Jinhua.

We integrated the external population with each indigenous breed to conduct the structure analysis, and the results showed that the reference panel could explicitly identify the commercial breeds, including D, L, and Y (Figure 4a), while several outliers might be caused by mislabeling.

Although the other European domestic breeds, including P, H, and B, were not included, the reference panel showed a moderate performance of ancestry identification (Figure 4a). The results showed that the P, H, and B breeds contained high EUD proportions, consistent with the pattern of the PCA (Figure S3). For instance, the Berkshire breed contained a higher Landrace proportion, and the Berkshire breed was also closer to the Landrace breed in PCA (Figure S3a). These results demonstrated the robust performance of ancestry identification for the reference panel.

The results also showed good ancestry identification for crossbreeds (Figure 4a). To further explore the performance of ancestry identification for crossbreeds, we randomly selected 15 valid crossbred individuals and the results indicated good ancestry identification in crossbreeds (Figure 4b). For example, the crossbreed D × (L × Y) and L × Y demonstrated the coincident ancestry proportion of D/L/Y. In addition, the crossbreed D × DNXE also demonstrated regular ancestry proportion (about 50% of the Duroc proportion and about 50% of the indigenous breed proportion).

We found good identification performance for indigenous breeds as well (Figure 4a). To further investigate the identification performance for indigenous breeds, we also randomly selected 16 valid individuals (including 4 indigenous breeds) and the results demonstrated that most of the valid individuals performed stable identification (Figure 4c). These results demonstrated the reference panel possessed good performance for ancestry identification in pigs.

## 4. Discussion

In this study, we constructed an ancestry reference panel and convenient pipeline for ancestry identification using PGRP v1. We applied population structure analysis (including PCA and structure analysis) to select reliable individuals of commercial/indigenous breeds. Then, we assessed the performance of ancestry identification of reference panel for EUD/ASD proportion and breed proportion. The results demonstrated that the reference panel performed excellently in ancestry identification, especially among crossbred individuals.

### 4.1. The Population Structure Analysis Could Estimate the Ancestry/Breed Proportion

The genotype data could be utilized to identify the ancestry proportion for pigs. The previous studies showed that the selection of tag SNPs using machine learning methods [13,14] could favorably identify the breed information for pigs. In addition, the population genetic analysis and machine learning model also demonstrated the effective classification for the breed information in eight chicken breeds [11]. However, these methods mainly focused on the classification of pure breeds. Previous studies have demonstrated the introgression from commercial breeds to indigenous breeds because of natural and artificial selection [24,25]. Some studies have also identified the potential selective regions for indigenous breeds, such as Jinhua pigs [26] and Duroc pigs [27]. These studies indicated that it was important to estimate the ancestry information across the purebred and crossbred. The selection of AIMs could partially classify the ancestry proportion for pigs [16], while it was challenging to identity the breed composition of crossbreeds. In addition, although the large reference panel could feasibly identify the pig breed, it is relatively complex to subdivide the breed composition of crossbreeds [17]. A population structure analysis, such as ADMIXTURE [21], could utilize the genotype data to estimate the ancestry proportion of the samples. The previous research showed that it could estimate the breed composition using a low-density SNP panel in African crossbred dairy cattle [28], which provided a feasible scheme to classify the breed composition in pigs. In this study, we developed a pipeline of population structure analysis using admixture software to estimate the ancestry proportion for pigs. Our results showed that the reference panel possessed the power to identify the breed composition of crossbreeds (Figure 4b).

### 4.2. The Ancestry Reference Panel Could Estimate the Breed Composition for Application

The breed composition of the pig partially influences the meat quality, and indigenous breeds possess higher meat quality compared with commercial breeds. For example, the Laiwu pigs has extremely high intramuscular fat content, better meat color, and lower drip [29]. Transcriptomics Analysis of muscle also showed that the differential expression genes correlated with the meat quality in the Min pig [30]. In addition, the previous study showed that the hybridization of indigenous and commercial breeds could improve the meat quality and growth efficiency [7]. The majority of pigs in the pork market are crossbred. It is important to explore the breed composition of pork to partially evaluate the meat quality and decide the price of pork. Here, we constructed an ancestry reference panel to estimate the breed composition of pigs, which could help us to indirectly identify the quality of pork meat in application.

### 4.3. The Limitation and Perspective the Ancestry Reference Panel

Although the ancestry reference panel covered the majority of breeds across the world, some breeds, such as Berkshire, Hampshire, and other indigenous breeds, were not involved in the reference panel, which limited the estimation of the breed composition of the candidates, especially if the breed was not included in the reference panel. Therefore, it is necessary to expand the breed composition of the ancestry reference panel to estimate the ancestry proportion better. On the other hand, although the population structure analysis could estimate the ancestry proportion across the samples, it was challenging to pinpoint the attribution of the ancestry proportion. It is also necessary to develop a comprehensive workflow to pinpoint the attribution of estimated ancestry proportion and evaluate the reliability of the ancestry proportion.

## 5. Conclusions

The reference panel and the convenient pipeline were significant sources for tracing the breed composition of pigs and genetic research. The ancestry reference panel and the convenient pipeline are provided in https://github.com/SCAU-AnimalGenetics/Ancestry-identification (accessed on 20 April 2023). However, we also found that ancestry identification of the reference panel was limited by breed composition. Therefore, it is necessary to develop and expand the breed composition of the reference panel.

## Data Availability

All related data and analyzed pipeline produced or analyzed in this study are available from the corresponding authors upon reasonable request. The analysis pipeline can be found at https://github.com/SCAU-AnimalGenetics/Ancestry-identification (accessed on 20 April 2023).

## Supplementary Materials

The following supporting information can be downloaded at: https://www.mdpi.com/article/10.3390/ani14233489/s1, Figure S1: The PCA of selected individuals for each commercial breed. The colored circles and grey circles represented the selected and removed individuals for Duroc (a), Landrace (b) and Yorkshire (c), respectively. The numbers located in the figure title indicated the corresponding breeds and sample size. The numbers located in the brackets at axis title indicated the explained genetic variance; Figure S2: The structure analysis for reference panel. (a) The PCA of reference panel. Each point represents the individual. The number located in brackets indicated the explained genetic variance. (b) The structure analysis with K = 2 using ADMIXTURE for the reference panel. EUD: European domestic breed, ASD: Asian domestic breed; Figure S3: The PCA for Berkshire (a), Hampshire (b) and Pietrain (c) with Duroc, Yorkshire and Landrace. The numbers located in the figure title indicated the corresponding breeds and sample size. The numbers located in the brackets at axis title indicated the explained genetic variance; Table S1 The brief information of the reference panel, Table S2 The results of structure analysis for indigenous Chinese breeds.
