# Supplementary material for: Evaluation of the Breed Composition of Pork via Population Structure Analysis in Pigs
Source: Animals (Basel). 2024 Dec 3;14(23):3489. doi: 10.3390/ani14233489 (PMC11639829; doi:10.3390/ani14233489)

Supplementary figures

Figure S1 The PCA of selected individuals for each commercial breed. The colored circles and grey circles represented the selected and removed individuals for Duroc (a), Landrace (b) and Yorkshire (c), respectively. The numbers located in the figure title indicated the corresponding breeds and sample size. The numbers located in the brackets at axis title indicated the explained genetic variance.

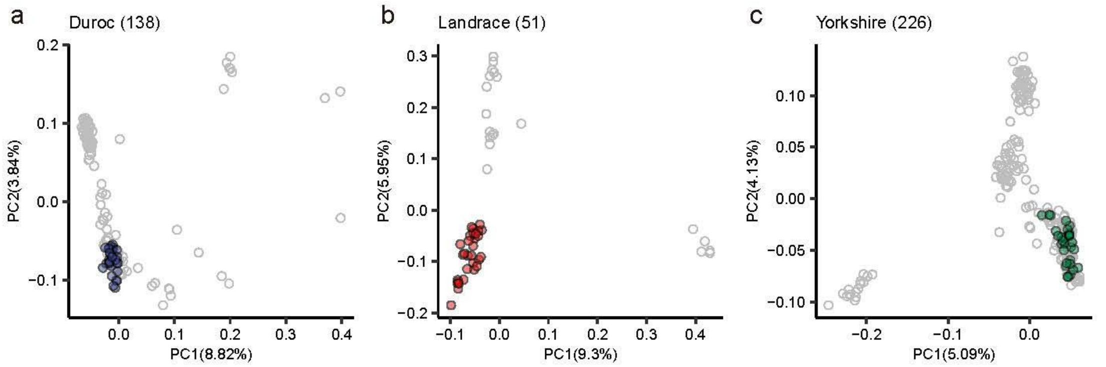

Figure S2 The structure analysis for reference panel. (a) The PCA of reference panel. Each point represents the individual. The number located in brackets indicated the explained genetic variance. (b) The structure analysis with K =2 using ADMIXTURE for the reference panel. EUD: European domestic breed, ASD: Asian domestic breed.

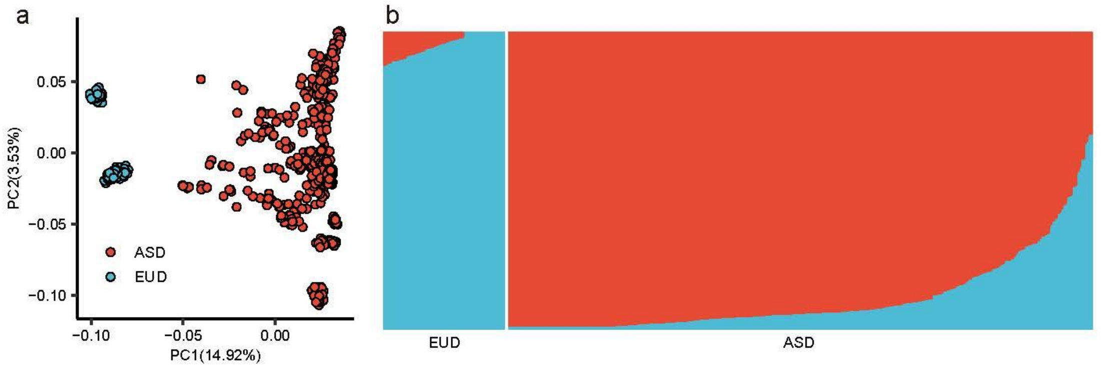

Figure S3 The PCA for Berkshire (a), Hampshire (b) and Pietrain (c) with Duroc, Yorkshire and Landrace. The numbers located in the figure title indicated the corresponding breeds and sample size. The numbers located in the brackets at axis title indicated the explained genetic variance.

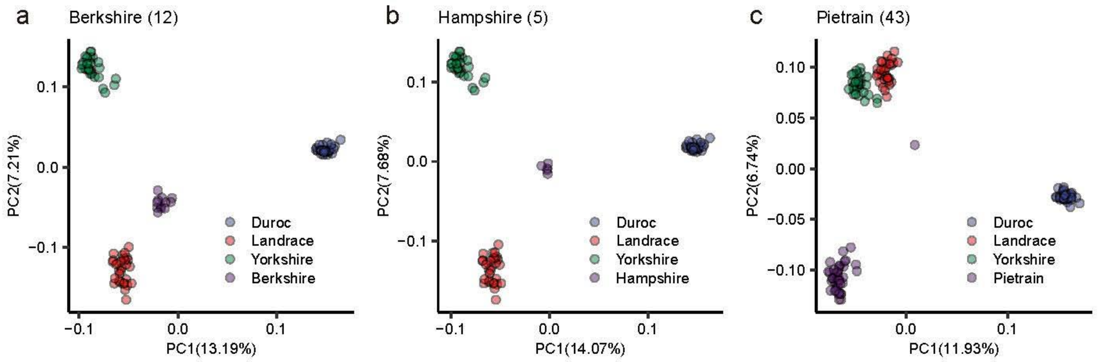

Supplement: Supplementary file 1 [file animals-14-03489-s001.zip › Supplementary figures information.pdf]
