# Supplementary figures and images for: Evaluation of the Breed Composition of Pork via Population Structure Analysis in Pigs
Source: Animals (Basel). 2024 Dec 3;14(23):3489. doi: 10.3390/ani14233489 (PMC11639829; doi:10.3390/ani14233489)

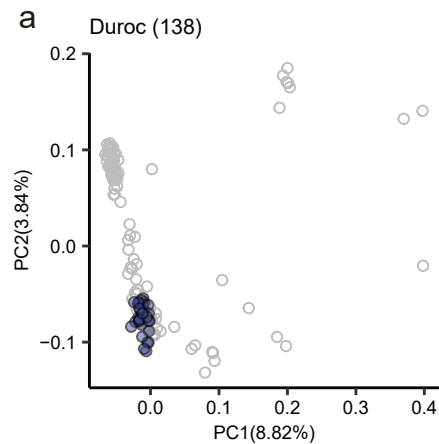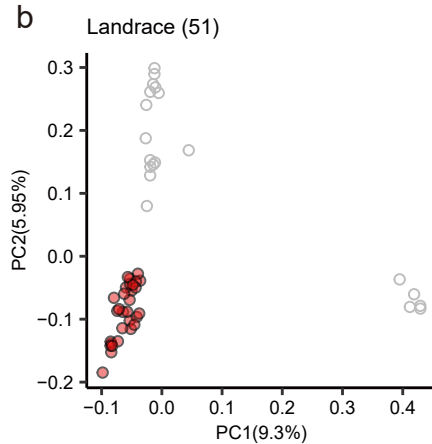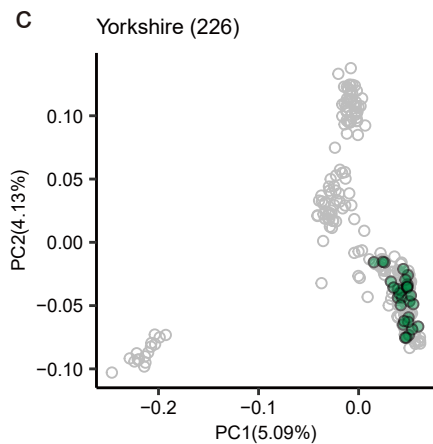

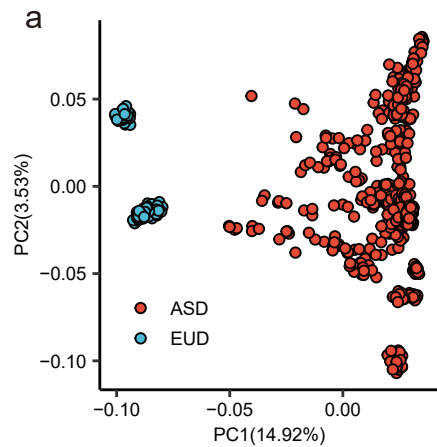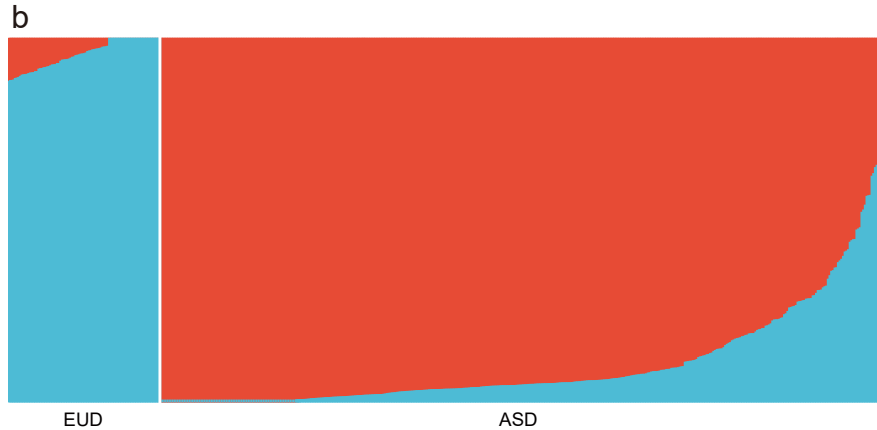

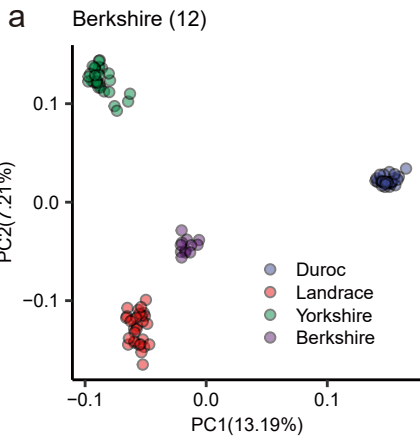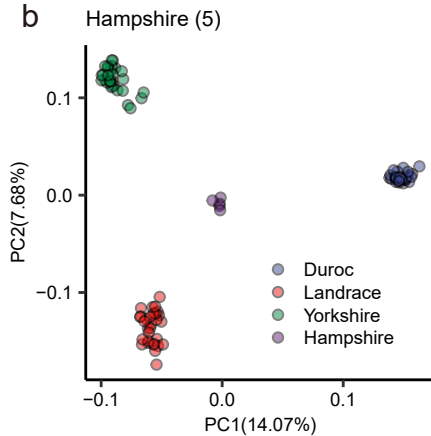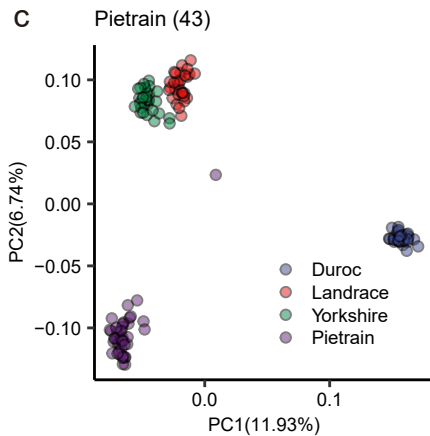

Supplement: Supplementary file 1 [file animals-14-03489-s001.zip › Supplementary figures.pdf]
